# Silent Rupture in the Inferior Vena Cava: A Complication Not to Be Ignored in the Presence of a Segment I Hydatid Cyst

**DOI:** 10.7759/cureus.53703

**Published:** 2024-02-06

**Authors:** Hicham Elmalki, Mehdi Moutawekkil

**Affiliations:** 1 Cardiothoracic Surgery Department, Laboratory of Anatomy, Microsurgery, and Surgery Experimental and Medical Simulation (LAMCESM), Faculty of Medicine and Pharmacy, Mohammed First University, Oujda, MAR

**Keywords:** inferior vena cava injury, complication, multidisciplinary, extracorporeal circulation, hydatid cyst

## Abstract

The presence of a cysto-caval fistula is a serious and rare complication of hepatic hydatid cysts, which can be life-threatening. We report a case of a 22-year-old patient with a segment I hepatic hydatid cyst discovered following scannographic imaging for non-specific abdominal pain. Management consisted of albendazole-based premedication for two weeks, followed by hepatic and venous resection surgery with prosthetic replacement after venous exclusion under extracorporeal circulation. To avoid hemorrhagic and/or embolic complications, it is essential to discuss rare cases of hydatid cysts with intimate contact or invasion of the vena cava in a multidisciplinary setting, to plan repair or reconstruction away from intraoperative surprises that are often fatal for benign pathology.

## Introduction

Hepatic hydatidosis is an endemic parasitic disease caused by accidental contamination of humans with *Echinococcus granulosus* [1]. It is often a silent and benign disease, with a risk of recurrence and complications that can be fatal [2]. Rupture into the inferior vena cava (IVC) is a rare and serious complication of hydatid cysts of the liver, which should be feared for any cyst with a cava contact regardless of its clinical picture [3], and surgical technique may go as far as hepatic exclusion, depending on radiological reconstruction and the data from surgical exploration [4]. We report the observation of a spontaneous intraoperative fistulization of a hepatic hydatid cyst in the IVC, which is rarely discussed in the literature and is without any real management consensus.

## Case presentation

We report a case of a 22-year-old patient from the Oriental region of Morocco, a farmer by profession with a history of contact with dogs. The patient was admitted for the management of a segment I hepatic hydatid cyst, discovered following non-specific abdominal pain that had been evolving for three months prior to admission. The patient had undergone a routine ultrasound in response to symptomatology, suggestive of a cystic image in the right liver, complemented by a CT scan showing the lesion.

**Figure 1:**
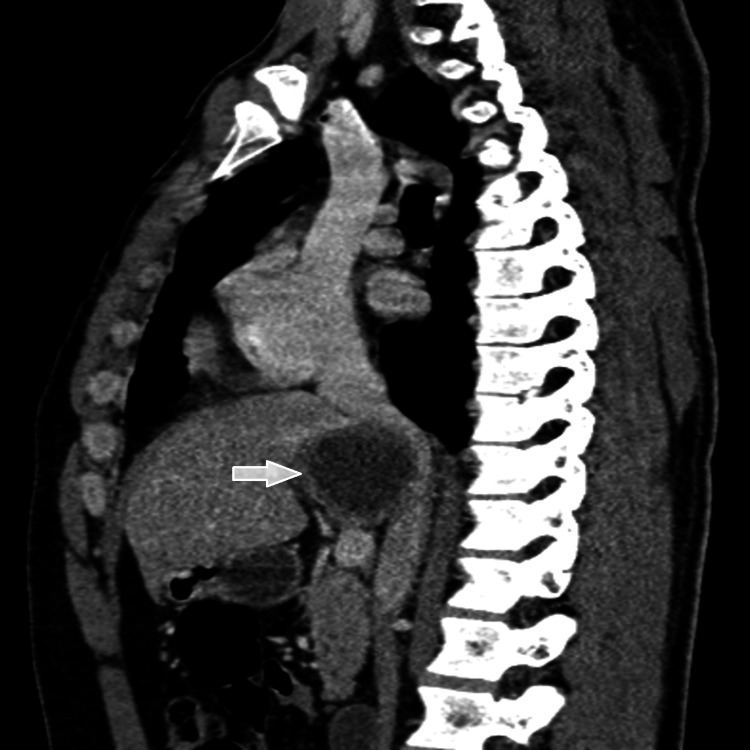
Sagittal reconstruction showing intimate contact with the inferior vena cava.

**Figure 2:**
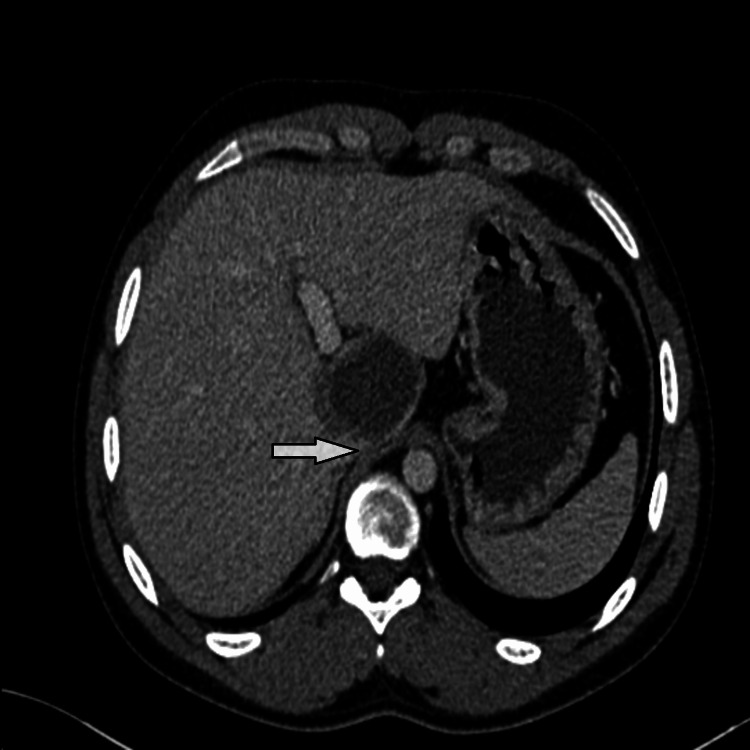
Horizontal section showing the wall of the vena cava caught by the cyst, indicating probable communication.

The lesion was located in segment I, was well limited, had a fluid content, with calcified wall in places, measured 59 x 45 mm versus 56 x 42 mm, and was in intimate contact with the IVC and the portal trunk (Figures 1, 2).

**Figure 3:**
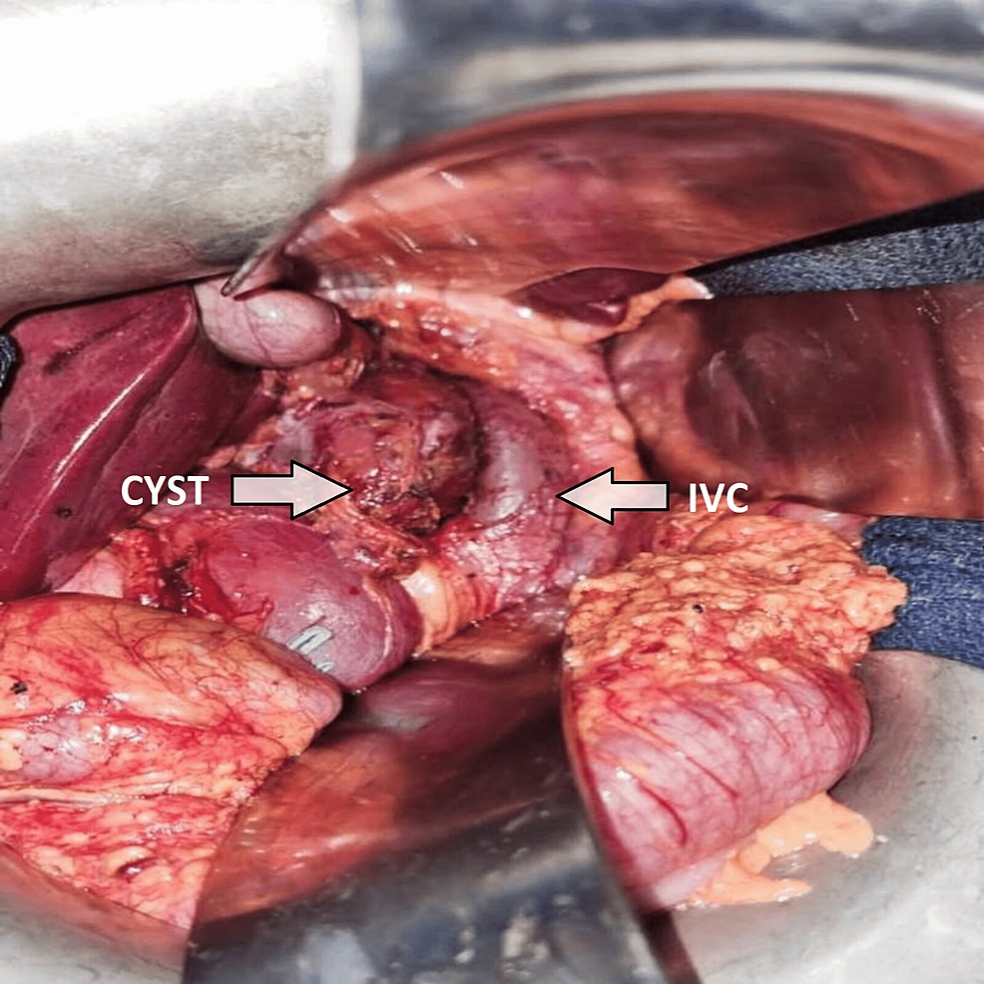
Perioperative image showing a cystic mass adjacent to the inferior vena cava (IVC).

**Figure 4:**
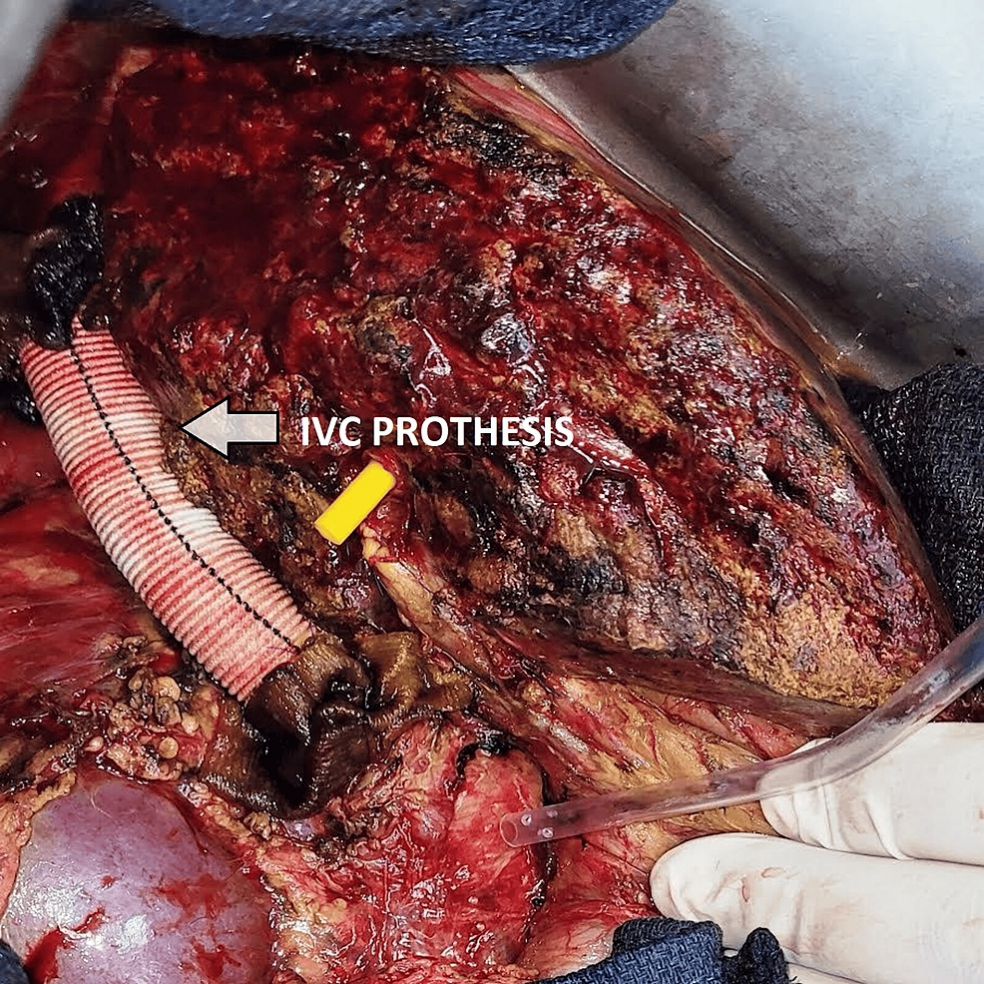
Final image after hepatic and vena cava resection and prosthetic replacement. IVC: inferior vena cava.

We completed the clinical history by requesting a hydatid serology test, which came back positive, and a hepatic and preoperative work-up, which came back without anomalies. Albendazole was prescribed 15 days prior to the operation, following a discussion of the case between several specialties involved in the management of the disease, which became imperative after vascular reconstruction had revealed cellar invasion. Surgery was performed under general anesthesia in three main stages: firstly, through a laparotomy, we controlled the retrohepatic IVC, which was invaded by the cyst, and then we proceeded to the vascular exclusion by placing a veno-arterial cardiopulmonary bypass through a median sternotomy. The arterial cannula was placed on the ascending aorta, the upper part of the body was drained by a snared superior vena cava cannula, and the lower body was drained through a right femoral vein cannula. The IVC was clamped above the renal veins and we placed a simple clamp on the portal trunk allowing hepatic and vena cava resection with minimal blood loss and without cystic rupture. Finally, a Dacron prosthesis was placed to reestablish lower venous cava return (Figures 3, 4).

**Figure 5:**
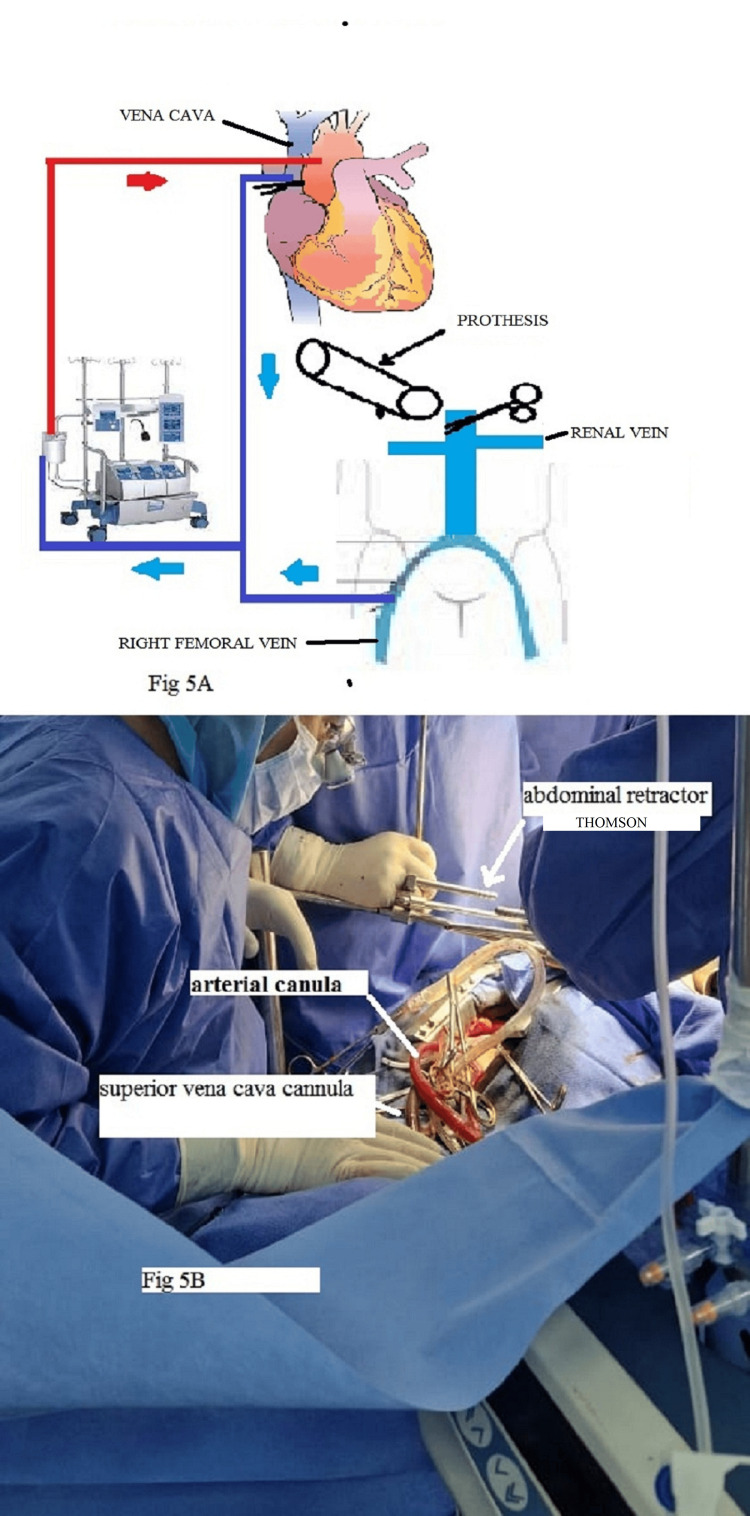
(A) Schematic drawing of the cardiopulmonary bypass. (B) Perioperative view.

A schematic drawing of the cardiopulmonary bypass is presented in Figure 5A. We focused on the hydatid cyst and the cava prosthesis, which is rare. The tip of the venous cannula in the right femoral vein was placed at the level of the right Scarpa triangle. When suturing the prosthetic graft, the IVC was clamped above the renal veins. The temperature of the extracorporeal circulation during IVC clamping was 32°C.

In this type of operation, it is necessary to use a short cannula since we need to resect the IVC, so we used a Medtronic Bio-Medicus 23 Fr femoral arterial cannula (Medtronic, Dublin, Ireland) in the femoral venous position with a vacuum in cardiopulmonary bypass. The position of the tip of the venous cannula was at the level of the iliac bifurcation. To avoid bleeding from the upper part of the IVC, we placed the first vent cannula in the pulmonary artery and the second vent cannula in the surgical field, which allowed us to perform the anastomosis in good conditions (Figure 5B).

The postoperative course was unremarkable, with no hemorrhagic or thromboembolic events, and the patient was discharged from the hospital on the 7th postoperative day. Our patient benefited from a three-month course of anti-parasitic medical treatment, with radiological control scheduled after six months.

## Discussion

Cystic echinococcosis is considered a rare disease in Europe, but it is endemic in some African and Asian countries, with significant financial costs [5]. The infection occurs accidentally in humans, and the hepatic location followed by the thoracic location is the most common [6,7]. It is often discovered fortuitously during a routine clinical or radiological examination [8], and sometimes in the presence of unusual or complicated clinical pictures [1]. Radiological diagnosis has been the subject of several publications and updates [9], but in some cases, it has not been possible to distinguish between a simple hydatid cyst or its differential diagnoses, such as cystadenoma or cystadenocarcinoma, until intraoperative view or on the basis of anatomopathological findings [10].

Despite the fact that hepatic localization is the most frequent, segment I cysts invading the IVC have rarely been discussed in the literature, and only isolated case reports are published without any real recommendations to follow [11]. This particular localization makes surgery for hydatid cysts, which is normally straightforward, highly complicated, sometimes necessitating vascular exclusion maneuvers [4]. The risk of flooding the operating field makes puncture of segment I cysts with cava contact inadvisable, and in most cases requires control of the IVC under and over the liver, thus, we used cardiopulmonary bypass to ensure a dry surgical field and to avoid a contamination risk [12]. Prosthetic reconstruction must be systematically anticipated and depends on preoperative reconstruction and intraoperative data, which allow judging the percentage of the circumference of the vena cava affected. Replacement appears mandatory when the invasion exceeds 180° [13]. This particularity of segment I hydatid cysts in the absence of codified consensus complicates the task of conventional postoperative follow-up [14], to which the complications of vena cava replacement are added [13,15].

## Conclusions

We report a new case of a segment I hydatid cyst of the liver invading the IVC. The particularity of our case lies in the need for preoperative radiological reconstruction coupled with multidisciplinary management. Hydatid cysts with cava contact should not be punctured until vascular control has been ensured. In some cases, exclusion from the vena cava with the use of temporary shunts to ensure venous return under extracorporeal circulation is essential, and protects the patient and the nursing team from life-threatening intraoperative complications.
